# Systemic and Retinal Protective Effects of Butyrate in Early Type 2 Diabetes via Gut Microbiota–Lipid Metabolism Interaction

**DOI:** 10.3390/nu17142363

**Published:** 2025-07-18

**Authors:** Haijun Gong, Haoyu Zuo, Keling Wu, Xinbo Gao, Yuqing Lan, Ling Zhao

**Affiliations:** 1 Guangdong Provincial Key Laboratory of Malignant Tumor Epigenetics and Gene Regulation, Department of Ophthalmology, Sun Yat-sen Memorial Hospital, Sun Yat-sen University, Guangzhou 510120, China; 2 State Key Laboratory of Ophthalmology, Guangdong Provincial Key Laboratory of Ophthalmology and Visual Science, Zhongshan Ophthalmic Center, Sun Yat-sen University, Guangzhou 510060, China; 3 Guangdong Provincial Key Laboratory of Brain Function and Disease, Guangzhou 510080, China

**Keywords:** diabetic retinopathy, butyrate, gut microbiota, lipid metabolism, WF SS-OCTA

## Abstract

**Background:** Early neurovascular unit (NVU) impairment plays a critical role in the pathogenesis of diabetic retinopathy (DR), often preceding clinically detectable changes. Butyrate, a short-chain fatty acid (SCFA) derived from gut microbiota, has shown promising metabolic and anti-inflammatory effects. **Methods**: This study investigated the protective potential of oral butyrate supplementation in a mouse model of early type 2 diabetes mellitus (T2DM) induced by a high-fat diet and streptozotocin. Mice (C57BL/6J) received sodium butyrate (5 g/L in drinking water) for 12 weeks. Retinal NVU integrity was assessed using widefield swept-source optical coherence tomography angiography (WF SS-OCTA), alongside evaluations of systemic glucose and lipid metabolism, hepatic steatosis, visual function, and gut microbiota composition via 16S rRNA sequencing. **Results:** Butyrate supplementation significantly reduced body weight, fasting glucose, serum cholesterol, and hepatic lipid accumulation. Microbiome analysis demonstrated a partial reversal of gut dysbiosis, characterized by increased SCFA-producing taxa (Ruminococcaceae, Oscillibacter, Lachnospiraceae) and decreased pro-inflammatory, lipid-metabolism-related genera (Rikenella, Ileibacterium). KEGG pathway analysis further revealed enrichment in microbial lipid metabolism functions (fabG, ABC.CD.A, and transketolase). Retinal vascular and neurodegenerative alterations—including reduced vessel density and retinal thinning—were markedly attenuated by butyrate, as revealed by WF SS-OCTA. OKN testing indicated partial improvement in visual function, despite unchanged ERG amplitudes. **Conclusions:** Butyrate supplementation mitigates early NVU damage in the diabetic retina by improving glucose and lipid metabolism and partially restoring gut microbial balance. This study also underscores the utility of WF SS-OCTA as a powerful noninvasive tool for detecting early neurovascular changes in DR.

## 1. Introduction

Diabetes mellitus (DM) is a metabolic disorder that primarily affects carbohydrate, lipid, and protein metabolism, especially glucose processing, due to either insufficient insulin production or decreased tissue sensitivity to insulin [1]. Diabetic retinopathy (DR), the most frequent and severe complication associated with DM, is the leading cause of blindness in the working-age population [2]. Despite considerable progress in the treatment of DR, early visual function impairments can occur even before the clinical onset of retinopathy [3]. Recent evidence has highlighted DR as a complex neurovascular disorder where neurovascular unit (NVU) deficits can precede vascular abnormalities in diabetic patients and animal models [4].

The interplay between lipid metabolism and gut microbiota has emerged as a key modulator of retinal health, particularly in the context of metabolic retinal diseases such as DR. The aberrant accumulation of lipid metabolites, including ceramides and free fatty acids, contributes to oxidative stress, mitochondrial dysfunction, and vascular inflammation in the retina [5]. In parallel, the gut microbiota has been recognized as a virtual organ, engaging in local interactions within the gut and establishing systemic connections with the retina to facilitate a range of physiological processes [6]. Recent evidence suggests that alterations in the gut microbiota composition may modulate host lipid metabolism through microbial-derived metabolites such as short-chain fatty acids (SCFAs), bile acids, and lipopolysaccharides [7]. Butyrate, a key SCFA derived from the microbial fermentation of dietary fibers, serves as an essential energy source for colonocytes, strengthens intestinal barrier integrity by upregulating tight junction proteins, and exhibits potent anti-inflammatory properties [8]. Notably, individuals with type 2 diabetes mellitus (T2DM) or prediabetes consistently exhibit a reduced abundance of butyrate-producing bacteria [9,10]. Although there is growing interest in the therapeutic potential of butyrate supplementation and gut microbiota modulation, the exact mechanisms by which they influence retinal lipid metabolism and vascular integrity—especially in the early stages of diabetes—remain unclear and require further investigation.

Traditional fluorescein fundus angiography, while standard for assessing early vascular changes, is invasive and has side effects, including anaphylaxis and nausea. Noninvasive, dye-free swept-source optical coherence tomography angiography (SS-OCTA) offers the three-dimensional visualization of retinal and choroidal NVU changes. Since the mid-peripheral retina is the primary capillary non-perfusion area, traditional low-field OCTA only focuses on the central retina and may miss important changes occurring in the mid-peripheral region. Widefield SS-OCTA (WFSS-OCTA), covering a broader peripheral retina (50–80°), allows for a better visualization of subtle early retinal changes [11,12]. However, no prior studies have evaluated NVU changes using WFSS-OCTA in experimental diabetes.

Based on emerging evidence linking gut microbiota, lipid metabolism, and retinal neurovascular health, we hypothesized that oral butyrate supplementation could protect against early neurovascular unit (NVU) impairment in type 2 diabetes mellitus (T2DM). In this study, we aimed to investigate whether butyrate exerts protective effects on the retinal NVU in a high-fat diet and streptozotocin (HFD/STZ)-induced diabetic mouse model. Specifically, we examined gut microbiota composition, systemic metabolic parameters (including glucose and lipid homeostasis), and local retinal outcomes. Furthermore, we employed widefield swept-source optical coherence tomography angiography (WF SS-OCTA) to noninvasively evaluate early retinal and choroidal microvascular and structural changes with and without butyrate intervention.

## 2. Materials and Methods

### 2.1. Experimental Animals and Diets

Male C57BL/6J mice aged six to eight weeks were sourced from the Gempharmatech Co., Ltd. (Nanjing, China). A T2DM mice model was established through a combination of a 60 kcal% high-fat diet (HFD) (D12492, Research Diets) for 4 weeks and a low-dose (30 mg/kg) streptozotocin (STZ) (Sigma-Aldrich, St. Louis, MO, USA, CAS#18883-66-4) intraperitoneal injection for 3 consecutive days (days 29–31). In order to determine the number of experimental mice, previous butyrate-related and ophthalmology studies were referred to [13,14].

**Figure 1:**
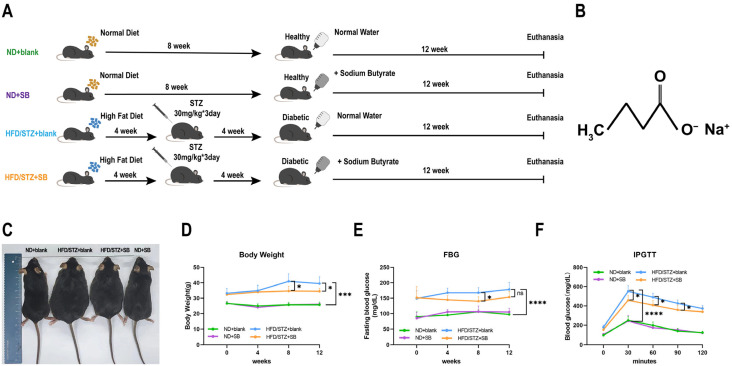
T2DM experimental animals exhibited metabolic disorders that were alleviated by butyrate. (**A**) Schematic diagram for the animal treatment. T2DM mice were constructed via a high-fat diet in conjunction with STZ injection. Sodium butyrate was administered for 12 weeks. (**B**) Molecular structure of compound sodium butyrate (SB). (**C**) Body scales of mice in different groups were displayed. (**D**) Body weights of mice were monitored at four-week intervals during the dosing period. (**E**) Fasting blood glucose (FBG) of mice was monitored at four-week intervals during the dosing period. (**F**) Intraperitoneal glucose tolerance test (IPGTT) with blood glucose measured every 30 min (*n* = 5 per group, one-way ANOVA test). ns: not significant, * *p* < 0.05, *** *p* < 0.001, ****p < 0.0001.

Male C57BL/6J mice (*n* = 20, 8 weeks old) were randomly divided into four groups (5 mice per group): normal diet (ND + blank), normal diet plus sodium butyrate (ND + SB), high-fat diet with STZ-induced diabetes (HFD/STZ + blank), and HFD/STZ plus sodium butyrate treatment (HFD/STZ + SB). Sodium butyrate (Sigma-Aldrich, CAS#156-54-7) was integrated into drinking water at a concentration for 12 weeks. Mice received sodium butyrate at 5 g/L in drinking water (~1 g/kg/day based on average consumption), a dose commonly used in previous metabolic studies in rodents [15]. Water intake was monitored, and no significant differences in average daily water consumption were observed among groups. The entire experimental procedure is visually represented in Figure 1A. The formula of sodium butyrate is C_4_H_7_NaO_2_, and its molecular structure is shown in Figure 1B. Animals were housed at standard laboratory conditions (25 °C, 12 h light–dark cycle) throughout the entirety of the 20-week experimental period. All animal experiments were approved by the Institutional Animal Care and Use Committee (IACUC) at Zhongshan Ophthalmic Center, Sun Yat-Sen University, and conducted in compliance with the UK Animals (Scientific Procedures) Act, 1986. Mice were fasted for 12 h prior to sacrifice in order to minimize variability in metabolic readouts. The mice from all experimental groups were subjected to the assessment of various parameters, including body weight, fasting blood glycemia, and their response to the intraperitoneal glucose tolerance test (IPGTT), which were measured using an Anwen + glucometer (Sinocare, Changsha, China).

### 2.2. Serum Lipid Parameters Evaluation

Throughout the experimental period, serum lipid parameters, including triglycerides (TGs), cholesterol (CHOL), low-density lipoprotein cholesterol (LDL-C), high-density lipoprotein cholesterol (HDL-C), apolipoprotein A1 (ApoA1), apolipoprotein B (ApoB), apolipoprotein E (ApoE), and lipoprotein (Lp), were regularly quantified using an automatic biochemical analyzer (AU5831, Beckman Coulter, Brea, CA, USA).

### 2.3. Hematoxylin–Eosin (HE), Oil Red O, Filipin III, Immunohistochemical (IHC) Staining

Liver and eyeball tissues were carefully harvested and processed for histological analysis using both paraffin embedding and cryosectioning techniques. For paraffin embedding, the tissues were fixed in 4% paraformaldehyde (Servicebio, Wuhan, China, G1109) at 4 °C overnight, followed by a graded ethanol dehydration series. The dehydrated samples were then embedded in paraffin. Hematoxylin counterstaining (BA4079, BASO, Zhuhai, China) was performed on the paraffin sections for general histological evaluation. For frozen sectioning, tissues were cryoprotected in sequential 20% and 30% sucrose solutions for 24 h after fixation, embedded in an optimal cutting temperature (OCT) compound, and sectioned at a thickness of 10 μm. Oil Red O staining solution (G1262, Solarbio, Beijing, China) was used to visualize lipid accumulation, and Filipin III (1:250 dilution, 70440, Cayman, Ann Arbor, MI, USA) was applied to the sections for 2 h at 4 °C to detect unesterified cholesterol. Immunohistochemical (IHC) staining was performed on both liver and retinal sections using primary antibodies against HMG-CoA reductase (HMGCR, 1:250 dilution, 13533-1-AP, Proteintech, Wuhan, China) and a low-density lipoprotein receptor (LDLR, 1:250 dilution, 10785-1-AP, Proteintech). Primary antibodies were incubated overnight at 4 °C and secondary antibodies were incubated at room temperature for 2 h after washing. Histological images were captured and evaluated under a light microscope at 200× magnification. Relative intensities were quantified using ImageJ 1.54g (NIH, Bethesda, MD, USA). To account for inter-sample variation, all images were captured under identical exposure and gain settings. The signal intensity was normalized to background fluorescence in adjacent non-stained regions within the same section.

### 2.4. 16S rRNA Sequencing, Classification, and Diversity Analysis

Fecal samples were aseptically collected into sterilized collection tubes, rapidly flash-frozen in liquid nitrogen, and subsequently stored at −80 °C. The extraction of total microbial DNA from fresh mouse feces was carried out utilizing the NanoDrop One (Thermo Fisher Scientific, Waltham, MA, USA). 16S rRNA genes of distinct regions (V3–V4) were amplified used specific primer (338F and 806R) with 12bp barcode. Sequencing libraries were generated using NEBNext^®^ Ultra™ II DNA Library Prep Kit for Illumina^®^ (New England Biolabs, Ipswich, MA, USA). The library quality was assessed on the Qubit@ 2.0 Fluorometer (Thermo Fisher Scientific, Waltham, MA, USA). At last, the library was sequenced on an Illumina Nova6000 platform and 250 bp paired-end reads were generated (Guangdong Magigene Biotechnology Co., Ltd. Guangzhou, Guangdong, China). Operational taxonomic unit (OTU) was one of the most common terms in microbiology. For each representative sequence, the silva (https://www.arb-silva.de/, accessed on 1 May 2025), RDP (http://rdp.cme.msu.edu/index.jsp, accessed on 1 May 2025), and Greengenes (http://greengenes.lbl.gov/, accessed on 1 May 2025) databases were used to annotate taxonomic information by search-syntax (set the confidence threshold to default to ≥0.8). The taxonomy of the species annotation was divided into seven levels: kingdom, phylum, class, order, family, genus, and species. Differentially enriched Kyoto Encyclopedia of Genes and Genomes (KEGG) pathways were identified based on OTUs in the different groups.

### 2.5. WFSS-OCTA Examination

To evaluate in vivo alterations in retinal morphology and vessel density, swept-source OCT angiography (SS-OCTA) was performed using the Micron IV system (BM-400K BMizar, Toward Medical Technology, Wuxi, China). Widefield (WF) SS-OCTA imaging was conducted at weeks 8 and 12 after treatment initiation to detect early neurovascular unit (NVU) impairment. Mice were anesthetized via the intraperitoneal injection of pentobarbital sodium (40 mg/kg), and pupil dilation was achieved using tropicamide–phenylephrine eye drops (Santen Pharmaceutical Co., Ltd., Osaka, Japan). High-quality images (signal strength ≥ 6/10) with a scan area of 24 mm × 20 mm were selected for analysis. Vessel densities were quantified in the superficial and deep retinal plexuses, choriocapillaris plexus (CCP), choroidal Sattler’s and Haller’s layers (CSHLs), iris, and the peripapillary retinal nerve fiber layer (pRNFL). Additionally, segmented ocular layer thicknesses were measured, including the inner retina (from the RNFL to the inner nuclear layer), the outer retina (from the IS/OS junction to the retinal pigment epithelium [RPE]), pRNFL, ganglion cell map (GMA), and cornea.

### 2.6. Optokinetic Nystagmus (OKN) Examination and Electroretinography (ERG) Test

Visual acuity was assessed by optokinetic nystagmus (OKN), measuring the maximum spatial frequency (cyc/deg) as mice tracked high-contrast rotating stripes (12°/s) and a black and white stripe contrast of 99.72% in a closed chamber. Each eye was tested separately under standardized clockwise/counterclockwise conditions, with eye movements monitored by a top-mounted camera.

ERG recordings were performed after 12 weeks of butyrate treatment using the Espion V6 system (Diagnosys, Lowell, MA, USA) following overnight dark adaptation. Mice were anesthetized, pupils dilated, and recordings obtained bilaterally via ring electrodes. ERG responses were obtained with light intensities of 0.01, 0.1, 1, and 10 cd·s/m^2^ under dark-adapted conditions and light intensities of 3, 10, and 30 cd·s/m^2^ under light-adapted conditions. Throughout each recording session, the mice were positioned on a temperature-regulated heating pad.

### 2.7. Statistics Analysis

Data are shown as the mean ± standard error of the mean. Significant differences between the 2 groups were analyzed using a 2-tailed unpaired *t*-test performed with GraphPad Prism software 9.0 (GraphPad Software, La Jolla, CA, USA). If there were more than two groups, the Kruskal–Wallis rank sum test or one-way ANOVA was used. A *p* value less than 0.05 was considered statistically significant. Alpha diversity was applied in analyzing the complexity of microbiota species diversity. Principal components analysis (PCA) was performed to obtain principal coordinates and visualize complex and multidimensional data by the RStudio 1.1.456. Sample cluster analysis was performed as a UPGMA (unweighted pair-group method with arithmetic means) method to interpret the distance matrix. A sample distance heat map was drawn based on the result of combining the 9 algorithms by a vegan package in R software. Linear discriminant analysis (LDA) effect size (LEfSe) analysis was used to find the biomarker of each group based on the homogeneous OTU_table. LDA was used to evaluate the impact of significant species by setting an LDA score ≥ 3 and obtaining the biomarkers in different groups.

## 3. Results

### 3.1. Amelioration of Glucose and Weight Dysregulation by Butyrate in T2DM Experimental Animals

The HFD/STZ model effectively mimicked early-stage T2DM. Compared with normal mice, diabetic animals exhibited significant increases in body weight and fasting blood glucose (FBG), which were partially ameliorated by butyrate supplementation (Figure 1C–E). By week 8, butyrate notably reduced FBG levels; however, the effect diminished by week 12. Intraperitoneal glucose tolerance tests (IPGTTs) also confirmed improved glucose handling in butyrate-treated mice (Figure 1F), suggesting improvements in insulin sensitivity and glucose metabolism.

### 3.2. Mitigation of HFD-Induced Lipid Abnormalities by Butyrate Supplementation

**Figure 2:**
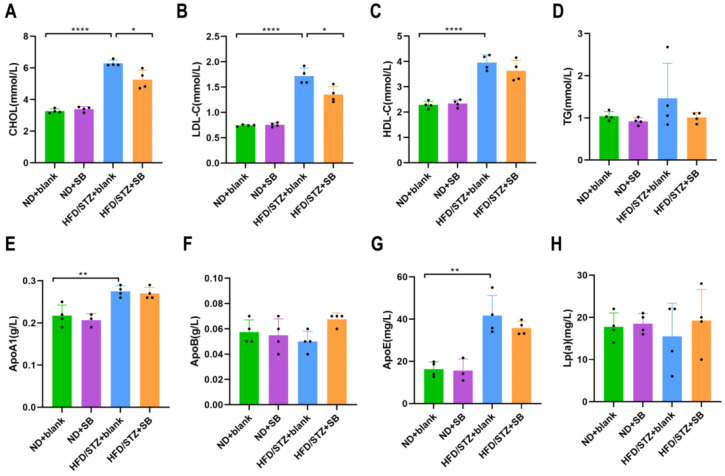
High-fat diet-induced serum lipid disorders that were reduced by butyrate diet: (**A**) CHOL (cholesterol), (**B**) LDL-C (low-density lipoprotein cholesterol), (**C**) HDL-C (high-density lipoprotein cholesterol), (**D**) TG (triglyceride), (**E**) ApoA1 (apolipoprotein A1), (**F**) ApoB (apolipoprotein B), (**G**) ApoE (apolipoprotein E), and (**H**) Lp (lipoprotein) levels were measured in the four groups (*n* = 4 mice per group, one-way ANOVA test).* *p* < 0.05, ** *p* < 0.01, **** *p* < 0.0001.

Given the observed protective effects of butyrate against HFD-induced adiposity in T2DM mice, we further assessed lipid metabolism by measuring a panel of serum lipid markers, including TG, CHOL, LDL-C, HDL-C, ApoA1, ApoB, ApoE, and Lp. T2DM mice exhibited significantly elevated levels of CHOL, LDL-C, HDL-C, ApoA1, and ApoE compared to those on a normal diet (Figure 2B–E,G). Notably, butyrate supplementation effectively reduced CHOL and LDL-C levels in HFD/STZ mice (Figure 2A,B).

**Figure 3:**
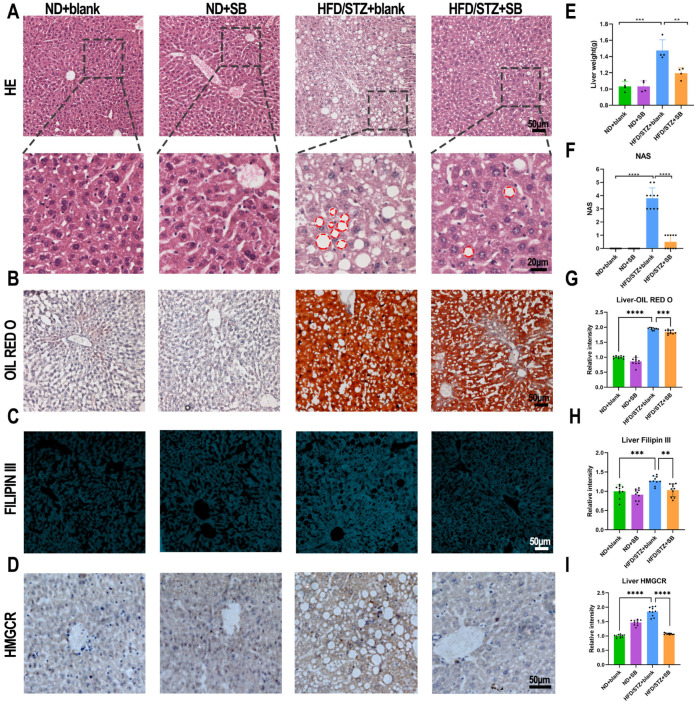
Hepatic steatosis and lipid accumulation in T2DM mice were alleviated by a butyrate diet. (**A**) Representative H&E-stained liver sections from the four groups show the overall hepatic architecture. Enlarged views of the boxed regions are provided below, with red circles highlighting lipid vacuoles within hepatocytes. (**B**–**D**) Representative images of Oil Red O staining, Filipin III staining, and HMGCR immunohistochemistry (IHC) demonstrate hepatic lipid accumulation and HMGCR expression across the groups. (**E**) Statistical comparison of liver weights among the four groups (n = 4 mice per group, one-way ANOVA test). (**F**) Quantitative analysis of non-alcoholic fatty liver disease (NAFLD) activity scores (NASs), indicating the severity of hepatic steatosis (*n* = 4 mice with 2–3 fields per mouse, one-way ANOVA test). (**G**–**I**) Quantification of Oil Red O-positive areas, Filipin III fluorescence intensity, and HMGCR IHC signal intensity in liver sections, respectively (*n* = 4 mice with 2–3 fields per mouse, one-way ANOVA test). Ns: not significant, ** *p* < 0.01, *** *p* < 0.001, **** *p* < 0.0001.

The liver serves as the primary site for CHOL and LDL production under both normal and diabetic conditions. HE staining revealed extensive lipid vacuole formation in the hepatocytes of untreated diabetic mice (Figure 3A), while Oil Red O and Filipin III staining demonstrated significant reductions in total hepatic lipid and CHOL accumulation, respectively, following butyrate treatment (Figure 3B,C,G,H). The NAFLD activity score (NAS) was calculated based on the Kleiner scoring system, comprising three components: steatosis (0–3), lobular inflammation (0–3), and hepatocellular ballooning (0–2). Liver sections were evaluated under light microscopy by two independent pathologists blinded to the group assignment, and the mean score was used for statistical analysis [16]. Consistent with a previous study showing that butyrate administration ameliorates hepatic fibrosis and steatosis in HFD-fed mice [17], we observed significantly increased liver weights and NASs in the HFD/STZ + blank group, which were substantially reduced following butyrate supplementation (Figure 3E,F). In addition, a clear downregulation of HMGCR expression in the liver was observed in the butyrate-treated group, suggesting reduced endogenous cholesterol production (Figure 3D,I).

**Figure 4:**
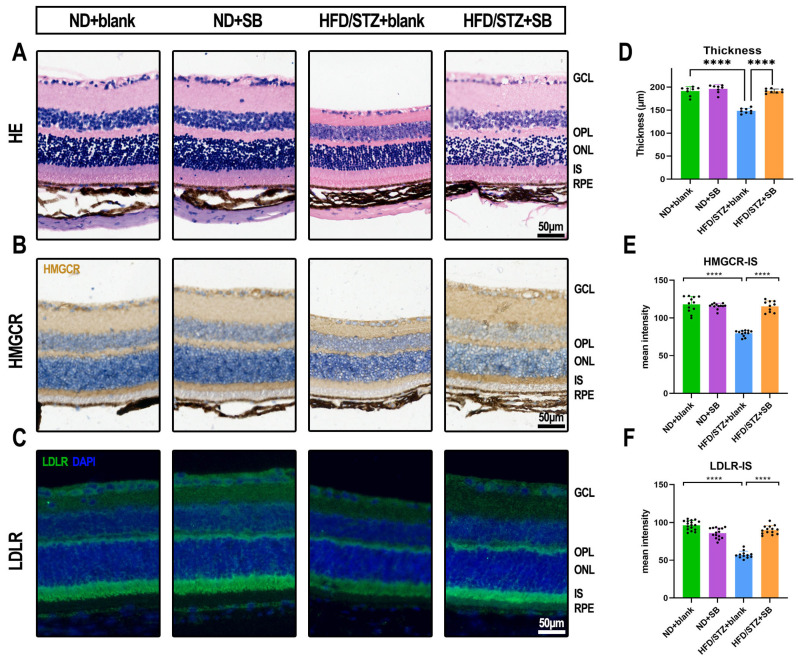
Butyrate protects the retina and enhances cholesterol metabolism in the retinas of type 2 diabetic mice. (**A**) Representative hematoxylin and eosin staining images illustrate the retinal structure across the four groups. Scale bar: 50 μm. (**B**) Representative immunohistochemistry images display retinal HMGCR expression in the four groups. (**C**) Representative immunofluorescence images show retinal LDLR expression in the four groups. (**D**) Quantitative analysis of retinal thickness in the four groups (*n* = 4 mice with 2 fields per mouse, one-way ANOVA test). (**E**) Quantification of mean HMGCR expression intensity in the inner retinal segments (*n* = 4 mice with 3 fields per mouse, one-way ANOVA test). (**F**) Quantification of mean LDLR expression intensity in the inner retinal segments (*n* = 4 mice with 3 fields per mouse, one-way ANOVA test). Scale bar: 50 μm. Ns: not significant, **** *p* < 0.0001.

Importantly, butyrate administration effectively mitigated retinal thinning in the inner and middle retinal layers of T2DM mice (Figure 4A,D). Immunohistochemical analysis showed a significant reduction in HMGCR expression within the photoreceptor inner segment layer of diabetic mice, which was notably reversed by butyrate supplementation (Figure 4B,E). In normoglycemic controls, LDLR was primarily localized to the inner segments (IS) and outer plexiform layer (OPL). However, in HFD/STZ mice, LDLR expression was substantially diminished in both regions. Remarkably, butyrate treatment restored LDLR expression to levels comparable to those observed in healthy controls (Figure 4C,F).

### 3.3. Butyrate Supplementation Partially Restored High-Glucose-Induced Gut Microbiota Dysbiosis and Modulated Its Interaction with Lipid Metabolism

**Figure 5:**
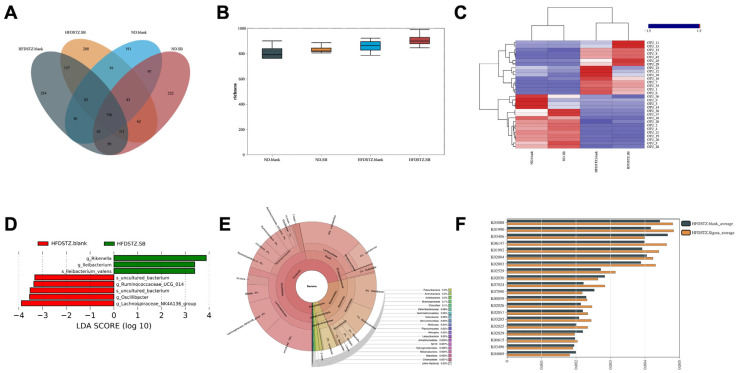
Partial restoration of high-glucose-induced gut microbiota dysbiosis and its interaction with lipid metabolism by butyrate supplementation. (**A**) Venn diagram displayed the OUT richness distribution in the four groups. (**B**) α diversity (Shannon index) based on the genus profile in the four groups was determined by one-way analysis of variance followed by Tukey’s post hoc tests. (**C**) Heatmap (log10) corresponding to their taxonomy at genus levels. (**D**) LDA scores (log10) for the most prevalent taxa in diabetic mice and butyrate supplements are represented on the positive scale, whereas negative LDA scores indicate enriched taxa in the diabetic controls (LDA > 3). (**E**) Krona chart illustrating the bacterial community structure based on 16S rRNA gene amplicon sequencing from a soil-water sample collected at a high-altitude cold desert lake. The hierarchical rings represent taxonomic levels from inner to outer circles: phylum, class, order, family, genus, and species. (**F**) KEGG pathways with differential enrichments of OTUs in the two diabetic mice groups. *n* = 5 mice per group.

A total of 2541 OTUs were clustered from high-quality reads at 97% sequence identity. The Venn diagram illustrated the distribution of OTU richness across the four experimental groups (Figure 5A). Alpha diversity, which reflects species richness within individual samples, showed no significant differences among the groups based on the Shannon index (Figure 5B). In contrast, beta diversity analysis—used to assess compositional differences between groups—revealed distinct clustering types in the heatmap based on genus-level taxonomy (Figure 5C). To further investigate microbial shifts after butyrate diet, we used LEfSe to identify differentially abundant taxa between the two diabetic groups. The relative abundance of *Rikenella* and *Lleibacterium* associated with host lipid metabolism and inflammatory responses was decreased, whereas SCFA productors—*Ruminococcaceae*, *Oscillibacter*, and *Lachnospiraceae*—were significantly increased after butyrate supplementation (Figure 4D). The bacterial cladogram at the OTU level also highlighted a distinct dominant lineage in HFD/STZ +SB mice (Figure 5E). To investigate the functional potential of the gut microbiota, we identified twenty KEGG pathways with differentially enriched OTUs. Among them, five pathways—including K00059 (fabG), K02003 (ABC.CD.A), K02004 (ABC.CD.P), K01192 (ribulose-5-phosphate 3-epimerase), and K01990 (transketolase)—were associated with lipid metabolism in this diverse environment (Figure 5F).

### 3.4. Early Neurovascular Alterations Are Attenuated by Butyrate: Evidence from WF SS-OCTA and OKN

**Figure 6:**
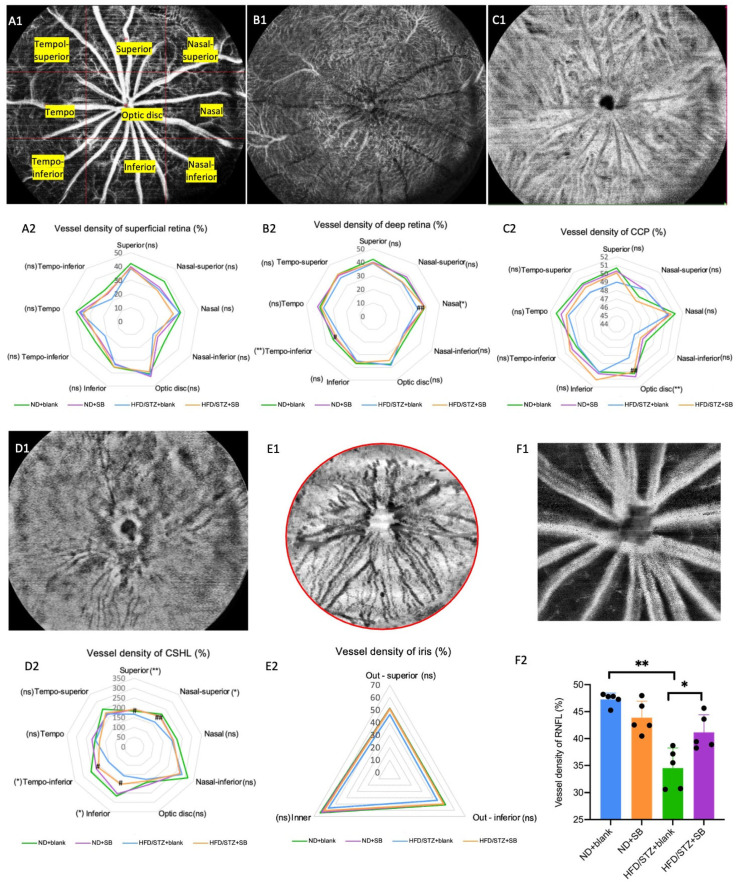
WFSS-OCTA detected vascular density changes in the T2DM mice. 3 × 3 grids (comprising nine rectangles: tempo-superior, superior, nasal-superior, tempo, optic disc grid, nasal, tempo-inferior, inferior, and nasal-inferior) with a total area of 24 mm × 24 mm of retina–choroid, iris, and pRNFL en face OCTA images were chosen to analyze the vessel density parameters. Representative OCTA en face images and statistical analysis showing the superficial retina (**A1**,**A2**), deep retina (**B1**,**B2**), CCP (**C1**,**C2**), CSHL (**D1**,**D2**), iris (**E1**,**E2**), and pRNFL (**F1**,**F2**). *n* = 5 mice per group, one-way ANOVA test. ns: not significant, * *p* < 0.05, ** *p* < 0.01 (HFD/STZ + SB group compared with ND + blank group), ^#^ *p* < 0.05, ^##^ *p* < 0.01 (HFD/STZ + SB group compared with HFD + blank group).

**Figure 7:**
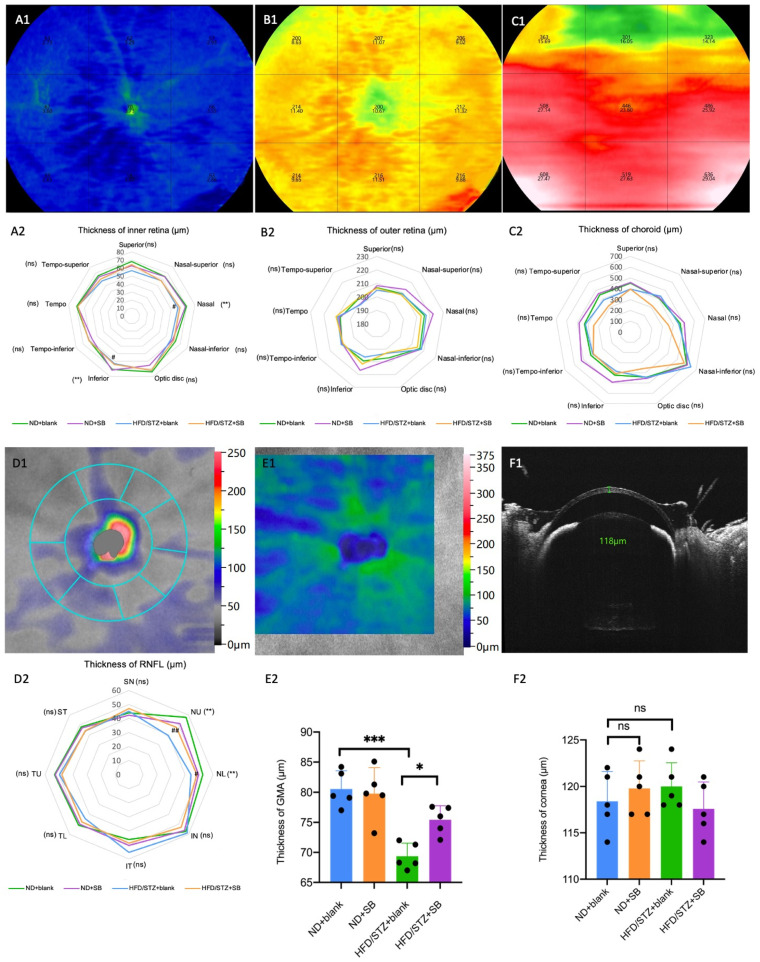
WFSS-OCTA detected ocular thickness alteration in the T2DM mice. 3 × 3 grids (comprising nine rectangles: tempo-superior, superior, nasal-superior, tempo, optic disc grid, nasal, tempo-inferior, inferior, and nasal-inferior) with a total area of 24 mm × 24 mm of retina–choroid, pRNFL, and GMA en face OCTA images were chosen to analyze the thickness parameters. Representative images and statistical analysis show the inner retina (**A1**,**A2**), outer retina (**B1**,**B2**), choroid (**C1**,**C2**), pRNFL (**D1**,**D2**), GMA (**E1**,**E2**), and corneal (**F1**,**F2**). *n* = 5 mice per group, one-way ANOVA test. ns: not significant, * *p* < 0.05, ** *p* < 0.01, *** *p* < 0.01 (HFD/STZ + SB group compared with ND + blank group), ^#^ *p* < 0.05, ^##^ *p* < 0.01 (HFD/STZ + SB group compared with HFD + blank group).

Using WFSS-OCTA, we assessed retinal and choroidal vessel density, as well as neurodegeneration, via pRNFL and GMA thickness. Hyperglycemia significantly reduced vessel density in the deep retina (nasal and tempo-inferior), CCP (nasal and optic disc), CSHL (superior, nasal-superior, tempo-inferior, and inferior), and pRNFL compared to the controls (Figure 6). Butyrate supplementation effectively preserved vessel density in these regions in HFD/STZ mice. Similarly, hyperglycemia reduced inner retinal (nasal, tempo-inferior, and inferior), pRNFL (NU and NL), and GMA thickness, while butyrate treatment mitigated these losses (Figure 7).

**Figure 8:**
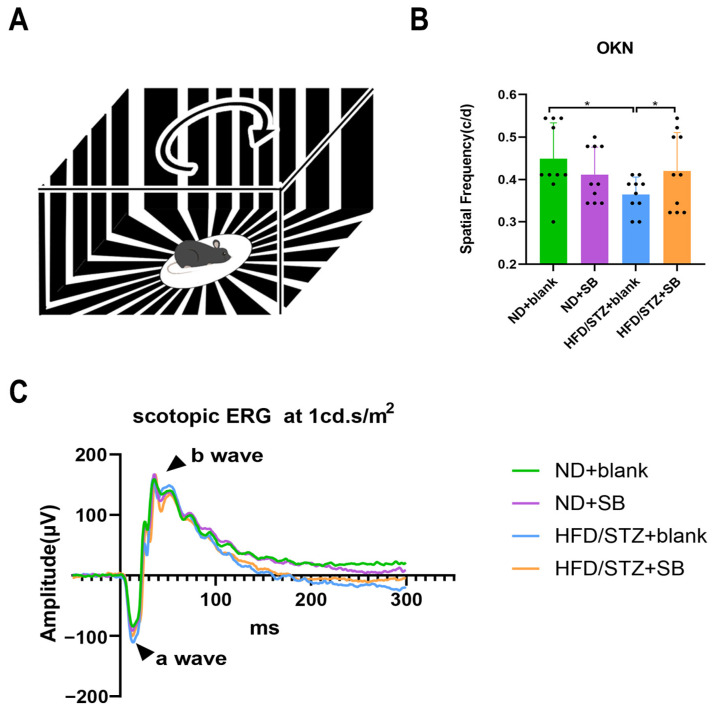
Butyrate improves visual function in type 2 diabetic mice. (**A**) Schematic diagram for the mice optokinetic nystagmus (OKN) test. (**B**) Statistical analysis for the OKN spatial frequency in four groups (*n* = 5 mice per group). (**C**) Representative scotopic ERG wave in four groups. Statistical analysis for the amplitude of B′ a-wave and B″ b-wave in scotopic ERG (*n* = 5 mice with 2 eyes per group, one-way ANOVA test). * *p* < 0.05.

Although dark-adapted ERG revealed no significant changes in a- or b-wave amplitudes post-treatment, OKN tests showed a slight decrease in visual perception in HFD/STZ mice, which was partially restored by butyrate supplementation (Figure 8).

## 4. Discussion

The primary feature of T2DM is the emergence of body insulin resistance that is marked by a decreased sensitivity in muscle, adipose, and hepatic tissues to this hormone. Consequently, it leads to a persistent state of chronic hyperglycemia [18]. In line with an earlier study [19], we hereby report that subjecting C57 mice to an HFD and intraperitoneal injections of STZ resulted in the development of obesity and prediabetes. We found that the administration of butyrate effectively mitigated all these alterations. Gao et al., in their study, proposed that the anti-obesity impact of butyrate stems from heightened body energy expenditure, the initiation of mitochondrial function, and increased fatty acid oxidation. These effects were mediated by the activation of PGC-1a in brown adipose and skeletal muscle tissues [20]. In addition, butyrate can activate free fat receptor 3, promote the release of the intestinal hormone peptide YY from endocrine cells, and increase the uptake of glucose in both muscle and adipose tissue [21].

Lipotoxicity primarily relates to impaired signaling and insulin resistance in non-adipose tissues, including the myocardium, pancreas, skeletal muscle, liver, kidney, and retina [22,23]. Pathogenic factors include CHOL, ceramide, and potential retinal-specific effects of fatty acid oxidation products, as well as lipid-independent effects of PPAR alpha activation. Butyrate serves a dual role in lipid metabolism: it functions as a substrate and also acts as a regulatory agent that influences lipid metabolism processes [24]. Li et al. demonstrated that butyric acid enhances fatty acid oxidation in brown adipose tissue, which, in turn, ameliorates obesity and insulin resistance induced by diet [25]. In our study, CHOL and LDL-C levels in the serum of HFD/STZ mice were effectively reduced by butyrate supplementation. Since LDL-C is generally regarded as the main carrier of CHOL, which transports CHOL from the liver to peripheral tissues in the blood, our results also indicate that butyrate can improve CHOL metabolism in HFD/STZ mice, especially by reducing the transport of hepatic CHOL to peripheral tissues. Consuming an HFD can lead to the development of a fatty liver, a condition commonly referred to as hepatic steatosis [26] and non-alcoholic steatohepatitis [27]. Interestingly, butyrate supplementation markedly alleviated liver steatosis by decreasing not only the number of hepatocytes affected (total liver weight and NAS) but also the size and frequency of cytoplasmic lipid droplets in our study.

The gut microbiota play a pivotal role in T2DM development [28]. Individuals with T2DM exhibited a reduction in butyrate-producing bacteria and an enhancement in microbial functions related to sulfate reduction and resistance to oxidative stress [29,30]. In our study, diabetic mice exhibited significant gut microbiota disruption, reflected by increased separation and diversity in the PCoA and heatmap analyses. Although butyrate supplementation did not significantly alter gut microbial α-diversity, it partially restored the microbial community composition, consistent with our observations and previous report [31]. In addition, confirming previous studies that demonstrated that butyrate regulated intestinal barrier function [32], we described herein a partial restoration of the gut microbiota after butyrate supplementation. LEfSe analysis demonstrated that the relative abundances of harmful *Rikenella* and *Illeibacterium* increased in the HFD/STZ mice. *Rikenella,* an anaerobic gut-residing bacterium associated with host lipid metabolism and inflammatory responses, is elevated in the gut microbiomes of leptin-resistant obese and diabetic mice [33]. *Illeibacterium*, a recently identified genus within the Firmicutes phylum, has been observed to fluctuate in abundance in response to dietary interventions such as high-fat or high-glucose intake. Its enrichment is often associated with increased intestinal permeability, low-grade inflammation, and metabolic disturbances [34]. Importantly, we observed that butyrate productors—*Ruminococcaceae, Oscillibacter*, *and Lachnospiraceae*—were significantly increased after diet supplementation.

Five KEGG pathways also reflected distinct microbial contributions to host lipid homeostasis. Specifically, fabG encodes 3-oxoacyl-[acyl-carrier-protein] reductase (K00059) [35], a key enzyme in bacterial fatty acid biosynthesis, potentially influencing host lipid absorption and synthesis. K02003 and K02004 are involved in ABC-type transport systems [36,37]. Moreover, K01192 and K01990, associated with the pentose phosphate pathway [38,39], could influence NADPH production and lipogenesis indirectly. These findings underscore the complex interplay between microbial metabolic functions and host lipid metabolism, suggesting that gut-microbiota-derived functional capacity may contribute to lipid dysregulation or restoration in metabolic disorders.

Our findings also demonstrate that hyperglycemia-induced microvascular and neurodegenerative alterations in the retina and choroid can be partially ameliorated by butyrate supplementation. Using WFSS-OCTA, we observed a significant reduction in vessel density in multiple deep retinal and choroidal regions, including the nasal and tempo-inferior sectors of the deep retinal capillary plexus, as well as the nasal and optic disc regions of the choriocapillaris. Our results are consistent with recent insights that retinal vascular and neural pathologies exist long before the development of clinically visible retinopathy [40]. In parallel, the hyperglycemia-induced thinning of inner retinal structures—including pRNFL and GMA—was attenuated by butyrate treatment, indicating its neuroprotective potential during early diabetic retinal neurodegeneration. While dark-adapted ERG showed no significant changes in retinal electrophysiology, the observed improvements in OKN-based visual perception further support functional preservation. Taken together, these data highlight butyrate’s dual role in maintaining vascular integrity and neuronal structure, underscoring its therapeutic promise in preventing early diabetic retinopathy-related complications. However, given that butyrate also has anti-glycemic, anti-lipotoxic, anti-inflammatory, and reinforced gut barrier activity in DM [8,41,42], this protective effect on the retina is difficult to be explained by single or multiple factors in our study.

Our study has several limitations. First, the relatively small sample size in each mouse group may have reduced the statistical power for detecting subtle changes, particularly in functional assessments such as ERG, potentially contributing to the negative findings in retinal electrophysiological responses. Baseline retinal vascular density was also not measured prior to treatment initiation in the current study. Second, the investigation focused on early-stage diabetes, characterized by mild retinopathy, without addressing advanced diabetic complications. It remains unclear whether butyrate exerts similar protective effects in the later stages of diabetic retinopathy. Third, fecal samples were collected only at the experimental endpoint, which limited our ability to track dynamic changes in the gut microbiota over time. Fourth, we did not evaluate the integrity of the intestinal epithelial barrier in diabetic mice or assess whether butyrate supplementation modulates this barrier. To address these limitations and further validate our findings, future studies should incorporate larger cohorts, multiple time points, and more comprehensive analyses, including molecular and histological assessments. Given that butyrate possesses antioxidant properties and may protect against STZ-induced pancreatic β-cell damage, improved glycemia could result from both enhanced insulin sensitivity and the preservation of β-cell function [17,43,44]. Future work incorporating insulin tolerance testing (ITT) and direct assessment of pancreatic islet integrity will help to clarify the respective contributions of these pathways. Although our results suggest a link between gut dysbiosis and retinal NVU impairment, the causal relationship remains to be clarified. Further investigations using fecal microbiota transplantation or targeted microbiota manipulation are warranted.

## 5. Conclusions

Our findings demonstrate that butyrate supplementation confers early protection against retinal neurovascular impairment in diabetes by modulating gut microbiota and improving systemic metabolic balance. WF SS-OCTA offers a promising, noninvasive tool for the early detection of NVU dysfunction in diabetic retinopathy, facilitating timely intervention strategies.

## Data Availability

The original contributions presented in the study are included in the article, further inquiries can be directed to the corresponding author.
